# Paeoniflorin attenuates cuproptosis and ameliorates left ventricular remodeling after AMI in hypobaric hypoxia environments

**DOI:** 10.1007/s11418-024-01781-7

**Published:** 2024-03-01

**Authors:** Xin Fang, Yaoxuan Ji, Shuang Li, Lei Wang, Bo He, Bo li, Boshen Liang, Hongke Yin, Haotian Chen, Duojie Dingda, Bing Wu, Fabao Gao

**Affiliations:** 1 Department of Radiology, West China Hospital, Sichuan University, No. 37 Guoxue Road, Chengdu, 610041 China; 2 Department of Radiology, Ningxia Medical University, Yinchuan, China; 3 Molecular Imaging Center, West China Hospital, Sichuan University, Chengdu, China; 4 School of Basic Medical Sciences, Yunnan University of Chinese Medicine, Kunming, China; 5 Department of Radiology, Yushu People’s Hospital, Yushu, Qinghai China

**Keywords:** Paeoniflorin, Acute myocardial infarction, Hypobaric hypoxia, Ventricular remodeling, Cuproptosis

## Abstract

This study investigates the cardioprotective effects of Paeoniflorin (PF) on left ventricular remodeling following acute myocardial infarction (AMI) under conditions of hypobaric hypoxia. Left ventricular remodeling post-AMI plays a pivotal role in exacerbating heart failure, especially at high altitudes. Using a rat model of AMI, the study aimed to evaluate the cardioprotective potential of PF under hypobaric hypoxia. Ninety male rats were divided into four groups: sham-operated controls under normoxia/hypobaria, an AMI model group, and a PF treatment group. PF was administered for 4 weeks after AMI induction. Left ventricular function was assessed using cardiac magnetic resonance imaging. Biochemical assays of cuproptosis, oxidative stress, apoptosis, inflammation, and fibrosis were performed. Results demonstrated PF significantly improved left ventricular function and remodeling after AMI under hypobaric hypoxia. Mechanistically, PF decreased FDX1/DLAT expression and serum copper while increasing pyruvate. It also attenuated apoptosis, inflammation, and fibrosis by modulating Bcl-2, Bax, NLRP3, and oxidative stress markers. Thus, PF exhibits therapeutic potential for left ventricular remodeling post-AMI at high altitude by inhibiting cuproptosis, inflammation, apoptosis and fibrosis. Further studies are warranted to optimize dosage and duration and elucidate PF’s mechanisms of action.

## Introduction

As a severe form of acute ischemic insult to the myocardium, acute myocardial infarction results from an abrupt and substantial reduction or occlusion of the blood flow to the coronary arteries and results in ischemic necrosis of the myocardial tissue. Despite numerous pharmacological interventions available for acute myocardial infarction (AMI) management, data from various health agencies highlight a persistent upward trend in early mortality rates associated with this condition [1]. Ventricular remodeling (VR), a process that takes place subsequent to an AMI, embodies the alterations both structural and functional, that transpire within the heart post-infarction. These changes are characterized by the thinning of the myocardium in the infarcted region and hypertrophy in the non-infarcted regions, accompanied by cardiac systolic dysfunction and a stimulated neurohormonal system. This results in tissue reconstruction, inflammation, and apoptosis, which can in turn trigger a series of pathophysiological changes that may eventually lead to heart failure [2]. In view of this, it is of paramount importance to delay and prevent VR after AMI to reduce the risk of heart failure (HF).

The heart is highly susceptible to hypoxic stress, and prolonged exposure to hypobaric hypoxia in high-altitude regions can impair myocardial structure and function. With increasing altitude and prolonged exposure, the imbalance between oxygen supply and demand will progressively exacerbate damage to the heart muscle [3]. Mitochondria are critical to cardiac energy metabolism, redox balance, calcium signaling, and inflammatory processes. Given these critical functions, mitochondrial therapies have emerged as promising strategies to address heart failure [4, 5]. Research has shown copper’s vital role in regulating mitochondrial respiratory chain activity, tricarboxylic acid cycle, and the production of reactive oxygen species (ROS) [6, 7]. Further, copper is recognized for its involvement in the pathophysiology of the cardiovascular system [8]. In particular, elevated copper concentrations have been consistently linked with HF and its prognosis [9].

*Paeonia lactiflora*, indigenous to China and northern Asia, holds a distinguished place among the six renowned flowering plants in China. With its vivid blooms earning it the sobriquet of the “flower of love”, its roots have historically been exploited for their inherent medicinal attributes [10]. Paeoniflorin, an occurring compound found in the roots of *Paeonia lactiflora*, has various beneficial effects on cardiovascular health. It exhibits pharmacological activities, such as preventing blood clotting, reducing the risk of thrombosis and atherosclerosis, fighting against cancer cells, dilating blood vessels, protecting against oxidative stress and inflammation, and regulating the immune system [10–12]. Numerous studies have underscored the advantageous effects of PF in reducing infarct size, improving hemodynamic parameters, and reducing the expression of Caspase-3 and Bax in rats subjected to ischemia/reperfusion injury [13]. PF has also been shown to regulate inflammatory cytokines such as tumor necrosis factor-alpha (TNF-α), (interleukin-6) IL-6, and (interleukin-10) IL-10, lower brain natriuretic peptide (BNP) levels, and suppress activation of caspase-3 and caspase-9 [14].

Given the importance of the potential protective effect of PF against left ventricular remodeling following AMI in a hypobaric hypoxic environment, investigation of this issue and the underlying mechanism cannot be overstated. However, there has been scant study on topic. Against this background, the current study investigated the effect of PF on ventricular remodeling in rats with AMI in a hypobaric hypoxic environment, with the aim of exploring the possible mechanisms of its action.

## Materials and methods

### Animal preparation

All experimental procedures were carried out adhering to the directives set forth by the Animal Care and Use Committee of the Animal Laboratory Center, located at the West China Hospital in Chengdu, China, with the ethical approval number No.20230731002. The care and management of the animals were conducted in strict adherence to the National Institutes of Health Guide for the Care and Use of Laboratory Animals, in addition to the guidelines set by the Association for Assessment and Accreditation of Laboratory Animal Care within the People’s Republic of China. A cohort of 90 specific pathogen-free (SPF) male SD rats, each weighing approximately 220 ± 10 g, was obtained from Sichuan Dashuo Biological Co., Ltd., located in Chengdu, China. Fifteen of these rats were accommodated for a duration of 12 weeks under standard conditions in Chengdu, at an altitude of 500 m, while the remaining 75 rats were similarly housed for 12 weeks in Yushu, Qinghai, which is situated at an altitude of 4,250 m. These environments were consistently upheld at an average temperature of 24 ℃, with a relative humidity ranging between 65 and 75%, and a light/dark cycle spanning 12 h. The rats were given unrestricted access to water and a standard diet.

Out of the initial 90 rats, myocardial infarction (MI) models were established in 60 rats by ligation at the origin of the left anterior descending coronary artery. The remaining 30 rats, which included 15 from the plain, were subjected to sham surgery. Thirteen rats succumbed, and 15 models were deemed unsuccessful, leading to the exclusion of 28 rats from the cohort. The remaining 62 rats were randomized into a plain sham operation control group (PSO control, *n* = 11), a hypobaric hypoxia sham operation control group (HSO control, *n* = 10), a MI model group (*n* = 11), and paeoniflorin (No. 23180–57-6, Meilunbio, Dalian, CHN) groups (PF groups, *n* = 30) segregated into high, middle, and low doses (9.00, 4.50, and 2.25 mg/kg body weight [BW]/d, with *n* = 10 in each group). The PSO control group, HSO control group, and the MI model group were administered 4 mL/kg/d of 0.9% saline solution.

### Cardiovascular magnetic resonance (CMR)

At the 4-week post-surgery mark, magnetic resonance imaging (MRI) was used to evaluate both cardiac structure and function in vivo for all rats. The imaging procedure was facilitated using a 7 T MRI system (provided by Bruker), equipped with a dedicated 4-channel rat cardiac coil designed for small-bore imaging. The measured parameters included left ventricular ejection fraction (LVEF), Left Ventricular Stroke Volume (LVSV), left ventricular end-diastolic volume (LVEDV), and left ventricular end-systolic volume (LVESV).

### Biochemical detection

After CMR at 4 weeks post-surgery, blood was collected via the abdominal aorta, and cardiac tissue samples were collected to assess bioindicators. Serum and tissue supernatants were obtained and analyzed using biochemical assays (Nanjing Jiancheng Bioengineering Institute) to quantify levels of copper (Cu), pyruvic acid, glutathione peroxidase (GSH-PX), malondialdehyde (MDA) and superoxide dismutase (SOD). Commercial ELISA kits (Elabscience Biotechnology) were used to measure inflammation-related factors including TNF-α, BNP, interleukin-1beta (IL-1β), and interleukin-18 (IL-18).

### Western blot analysis

Proteins were extracted from cells and tissues, including the treatment of suspended cells, adherent cells, and fresh tissues. The extraction process involved the use of RIPA lysis buffer, centrifugation, oscillation, and homogenization. This step was completed 4 weeks after performing the surgical model. Subsequently, the protein concentration was determined using the Bicinchoninic Acid (BCA) protein assay method. Approximately 50 µg of total protein was separated through 10% Sodium Dodecyl Sulfate–Polyacrylamide Gel Electrophoresis (SDS-PAGE). The samples were then transferred onto a Polyvinylidene Difluoride (PVDF) membrane. These membranes were further incubated with various primary antibodies for 12 h at 4 °C. Subsequently, the membrane was exposed to HRP-marked goat anti-rabbit (dilution 1:10000, 70-GAR0072, Multi sciences) or anti-mouse secondary antibodies (1:10000 dilution, 70-GAM0072, Multi sciences) for one hour at room temperature. The bands were scanned using the Odyssey dual-color infrared imaging system, and protein expression levels were normalized to β-Actin (1:5000 dilution, AF7018, Affinity). The primary antibodies used for incubation included GSDMD (1:1000 dilution, AF4012, Affinity), Bcl-2 (1:1000 dilution, AF6139, Affinity), HSP 70 (1:1000 dilution, AF5466, Affinity), Caspase-1 (1:1000 dilution, AF5418, Affinity), NLRP3 (1:1000 dilution, DF7438, Affinity), DLAT (1:1000 dilution, #12362, CST), Bax (1:1000 dilution, GB11690, Servicebio), and Ferredoxin 1 (1:3000 dilution, AB108257, Abcam).

### Histopathological analysis

After the 4th week following surgical modeling, rats were subjected to isoflurane anesthesia and their hearts were collected for examination subsequent to the CMR evaluation. After the harvest, the hearts underwent a 12-h incubation period in paraformaldehyde. Subsequently, they were dehydrated and embedded in paraffin wax. The tissues that were embedded were then sliced into 6-μm sections using a microtome. These sections were later stained with haematoxylin and eosin (H&E) for histological analysis to assess myocardial damage. To quantify the volume fraction of cardiac collagen (CVF, %), Masson’s trichrome stain was utilized. This stain effectively differentiated nuclei, cytoplasm, and collagen by coloring them as purple, red, and blue respectively.

### TdT-mediated dUTP nick end-labelling (TUNEL) staining

The extent of apoptosis was determined through terminal deoxynucleotidyl transferase-mediated dUTP nick end-labelling (TUNEL) staining, using a TUNEL kit supplied by Promega, USA. Cardiomyocytes that were positive for TUNEL staining were enumerated using ImageJ software.

### Transmission electron microscopy (TEM)

The apex of the left ventricle was dissected to obtain tissue samples, which were then fixed in a 2.5% glutaraldehyde solution for 4–5 h. Subsequently, the samples underwent an hour-long postfixation process using 1% osmium tetroxide, followed by dehydration through a series of acetone solutions with varying concentrations and embedding in Araldite. To visualize the ultrastructural details of organelles, vascular cells, and cardiomyocytes, we utilized the JEM-1400PLUS electron microscope (manufactured by JEOL, Japan). Images were captured using a JEM-1400FLASH camera (Japan), while mitochondrial damage was assessed using the Flameng scoring system.

### Statistical analysis

Quantitative data are presented as mean ± standard deviation (SD). Statistical comparisons between experimental groups were performed using one-way ANOVA followed by Tukey’s post-hoc test for multiple comparisons. GraphPad Prism 8.0 software was used for analyses. P values less than 0.05 were considered statistically significant.

## Results

### PF ameliorates cardiac injury in AMI rats under hypobaric hypoxia

**Fig. 1:**
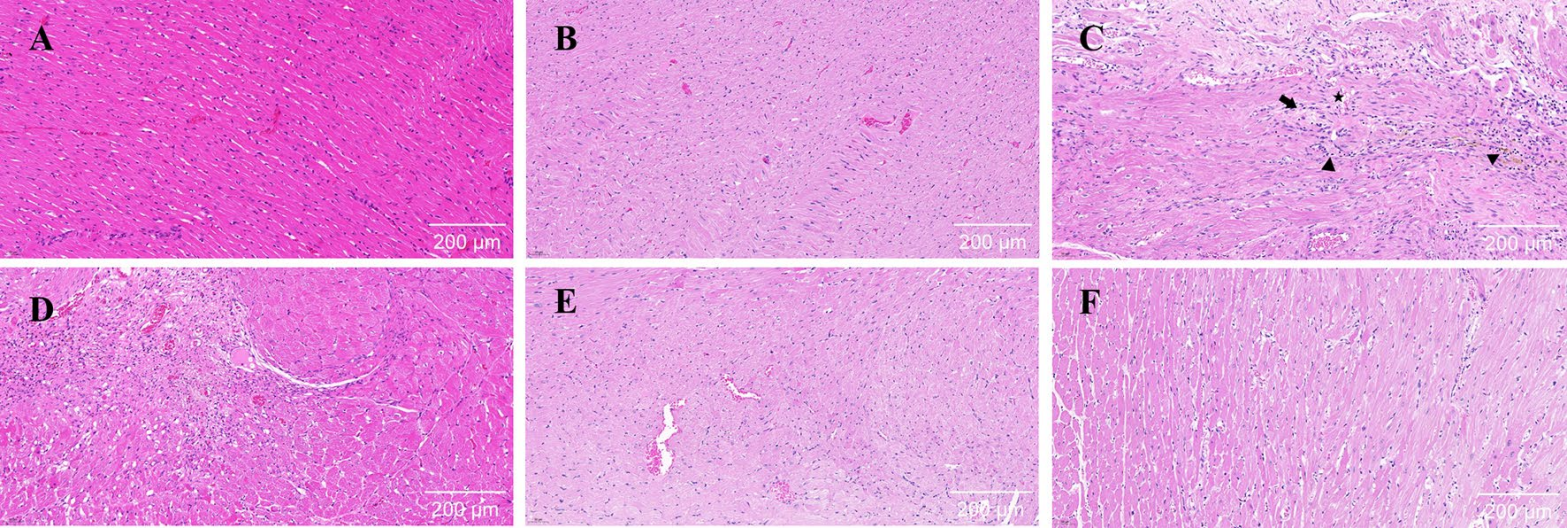
Representative sections of H&E-stained hearts (scale bar: 200 μm): **A** plain sham-operated control; **B** hypobaric hypoxia sham-operated control; **C** model control; **D** low-dose group; **E** middle dose group; **F** high-dose group. (Black arrow-myocardial necrosis; Black triangle-inflammatory cell infiltration; Black pentagram-myocardial telangiectasia)

Histological examination with H&E staining showed extensive focal myocardial infarction in the model group (Fig. 1). This was characterized by a degree of disruption and rupture of myocardial fibers, disturbance in the arrangement of some myocardial fibers, and nuclear contraction and fragmentation in some cells. There were also clear indications of myocardial telangiectasia and infiltration of inflammatory cells in the subepicardial region. In addition, necrosis of the myocardial tissue was clearly visible, but this was significantly reduced in the PF-treated rats.

**Fig. 2:**
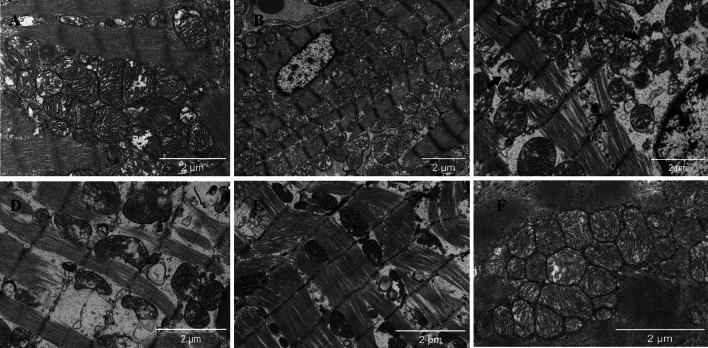
Representative transmission electron microscope images (scale bar: 2 μm): **A** plain sham-operated control; **B** hypobaric hypoxia sham-operated control; **C** model control; **D** low-dose group; **E** middle dose group; **F** high-dose group. (Black arrow-broken and dissolved mitochondrial cristae; Black pentagram-vacuolated mitochondria)

TEM of myocardial tissue showed ultrastructural abnormalities in cardiomyocytes such as edema and electron-lucent vacuoles, corroborating the protective impact of PF against AMI-induced damage. There was a notable increase in the number of collagen fibers, which exhibited a more tangled arrangement and wobbly structures. Some mitochondrial cristae were broken and dissolved, and the mitochondria showed signs of vacuolation or the formation of concentric hollow structures. However, these changes were alleviated after PF treatment, particularly in the group receiving the middle dose of PF (Fig. 2).

### PF ameliorates left ventricular dysfunction in AMI rats under hypobaric hypoxia

**Fig. 3:**
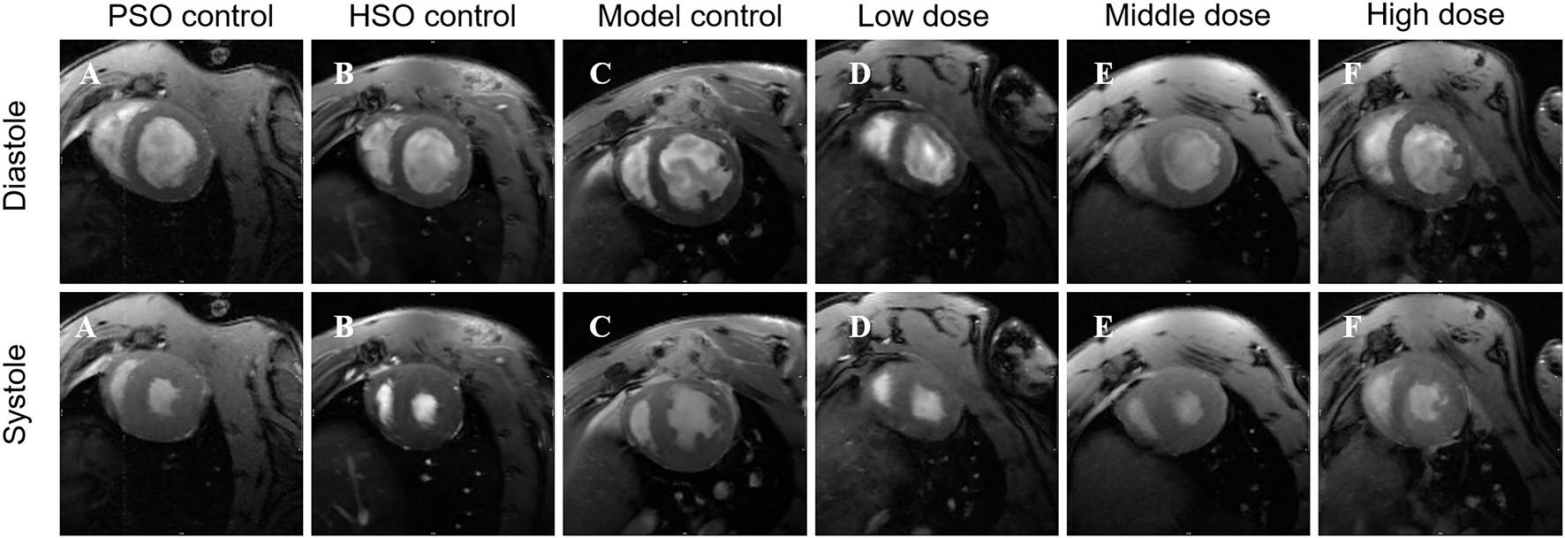
CMR images capturing the diastolic and systolic states of left ventricles were acquired from six distinct groups

CMR was conducted 4 weeks post-surgical modeling to evaluate the impact of PF on the cardiac function of rats subjected to hypoxia and hypoxia with AMI rats. Exemplary images are displayed in Fig. 3.

The model group exhibited a significant reduction in LVEF alongside a concurrent increase in both LVEDV and LVESV (Table 1). These changes suggest decreased cardiac efficiency and ventricular dysfunction, characteristic of heart failure: reduced LVEF indicates impaired heart pumping; increased LVEDV suggests ventricular dilation; and elevated LVESV points to inefficient blood expulsion. Interestingly, treatment with PF partially mitigated these alterations.

**Table 1:** Assessment of LV functional parameters 4 weeks post-surgery

Parameters	PSO	HSO	Model control	Low dose	Middle dose	High dose
LVEF (%)	70.69 ± 3.48	70.85 ± 1.55	45.80 ± 7.53###	57.96 ± 4.29**	65.91 ± 1.91 ***	62.09 ± 2.11***
LVEDV (mL)	0.69 ± 0.08	0.67 ± 0.08	0.94 ± 0.07##	0.77 ± 0.09*	0.78 ± 0.07*	0.71 ± 0.09**
LVESV (mL)	0.20 ± 0.03	0.20 ± 0.03	0.51 ± 0.08###	0.32 ± 0.03***	0.27 ± 0.04***	0.27 ± 0.04***
LVSV (mL)	0.49 ± 0.06	0.48 ± 0.06	0.43 ± 0.08	0.45 ± 0.08	0.53 ± 0.04	0.44 ± 0.06

The results represent the means ± SDs. *P < 0.05 and **P < 0.01 and ***P < 0.001 vs. the model control; #*P* < 0.05 and ##*P* < 0.01 and ###*P* < 0.001 vs. the PSO control

**Fig. 4:**
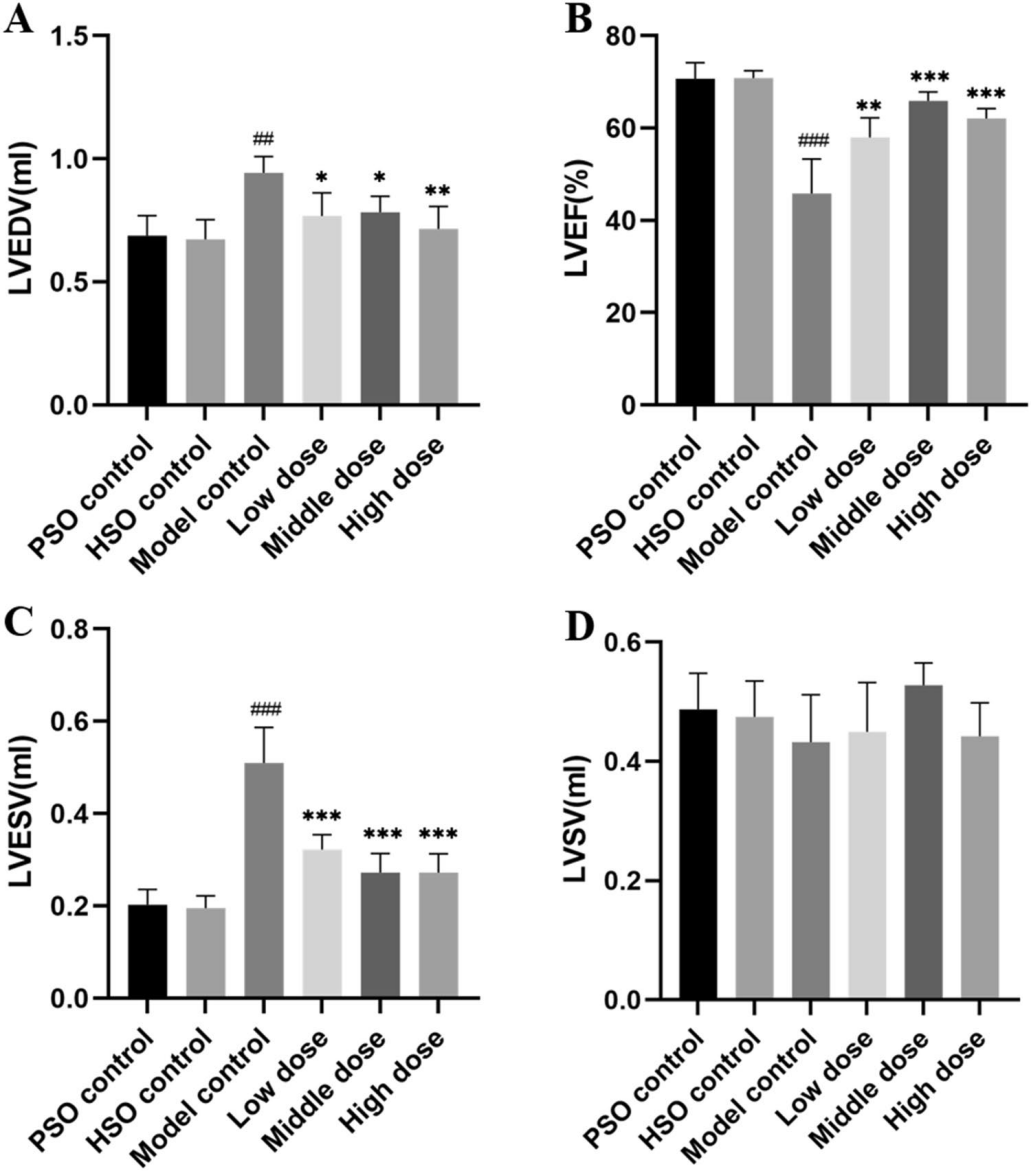
**A**–**D** Assessment of left ventricular function through measurement of the LVEF, LVEDV, LVESV, and LVSV. The values represent the means ± SDs. ^*^P < 0.05 and ^**^P < 0.01 and ^***^P < 0.001 vs. the model control; ^#^*P* < 0.05 and ^##^* P* < 0.01 and ^###^*P* < 0.001 vs. the PSO control

Furthermore, despite observing a decline in LVSV within the model group, this decrease did not reach statistical significance (Fig. 4).

### PF ameliorates cuproptosis in AMI rats under hypobaric hypoxia

**Fig. 5:**
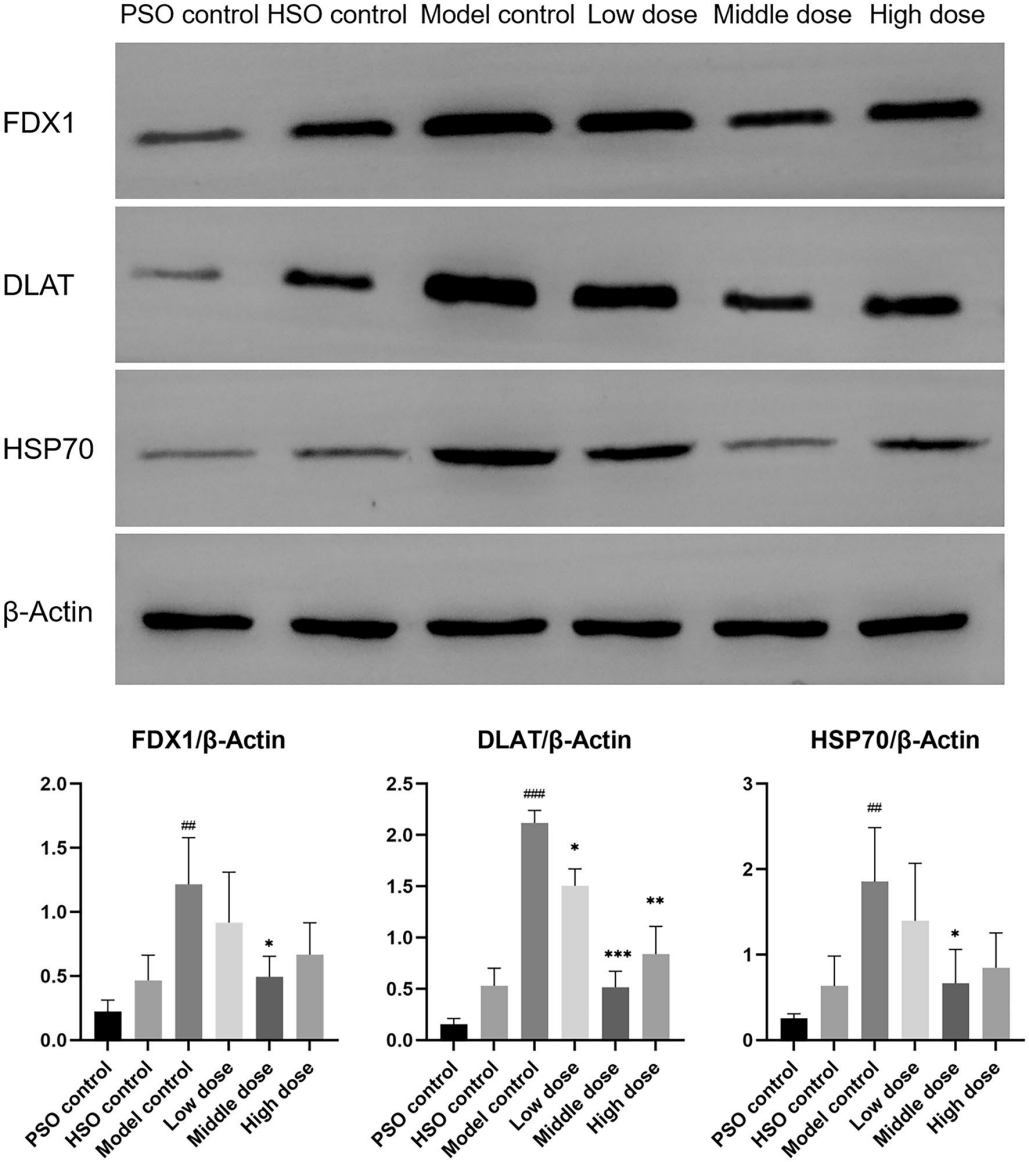
Western blots were performed to assess the protein levels of FDX1, DLAT, and HSP70, with corresponding statistical analysis presented. (*n* = 3). The values represent the means ± SDs. ^*^P < 0.05 and ^**^P < 0.01 and ^***^P < 0.001 vs. the model control; ^##^*P* < 0.01 and ^###^*P* < 0.001 vs. the PSO control

We examined the left ventricular expression of major cuproptotic markers FDX1, DLAT, and HSP70 by Western blot to assess the influence of PF on cuproptosis. PF treatment significantly mitigated cuproptosis induction compared to the model group, as evidenced by reduced FDX1, DLAT, and HSP70 levels (Fig. 5). These results demonstrate PF can attenuate deleterious cuproptotic signaling in the myocardium post-infarction, highlighting its therapeutic potential to combat copper-mediated cell death pathways.

**Fig. 6:**
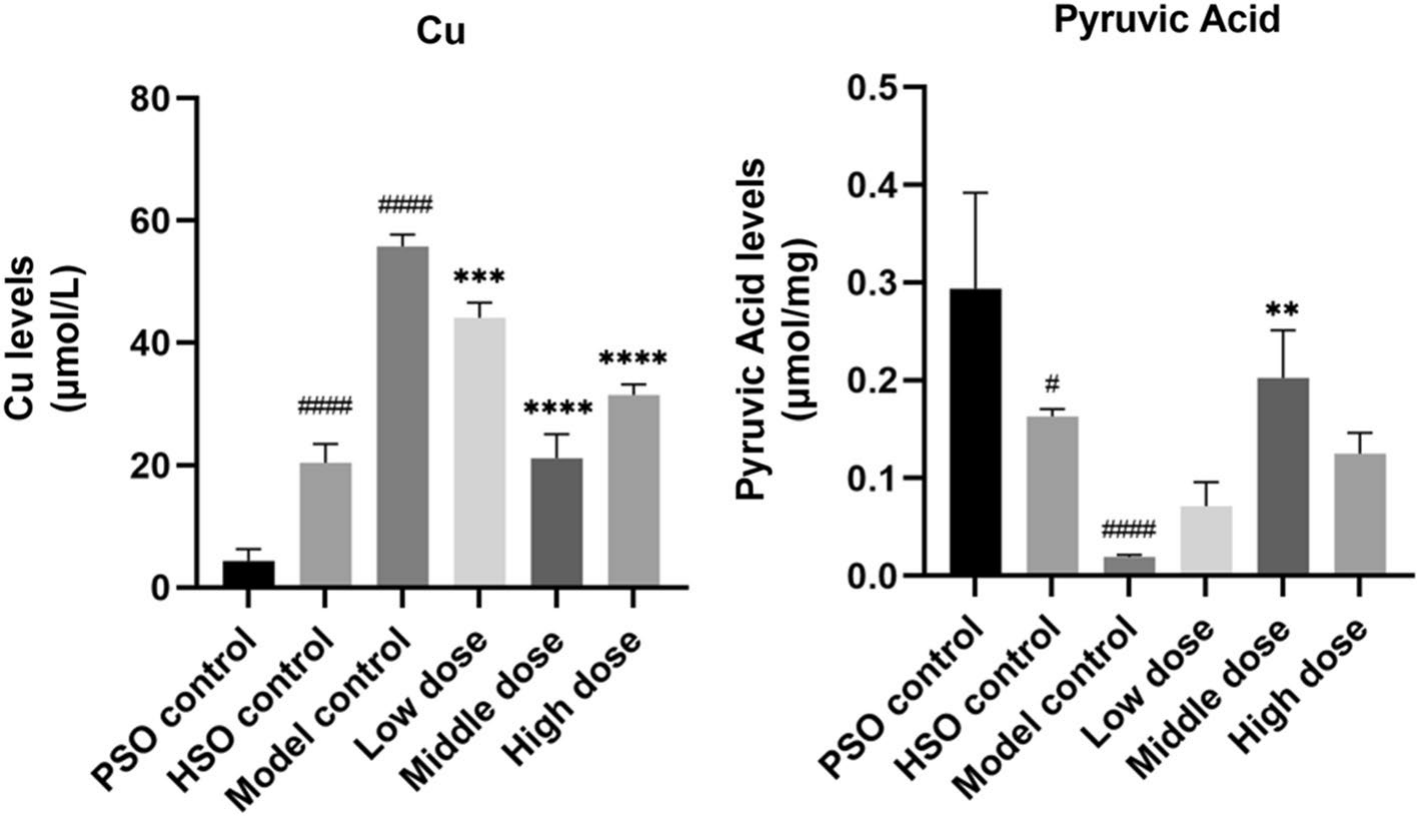
The serum levels of IL-1β, IL-18, and TNF-α, as measured by ELISA (*n* = 6). The values represent the means ± SDs. ^**^P < 0.01 and ^***^P < 0.001 and ^****^P < 0.0001 vs. the model control; ^#^*P* < 0.05 and ^###^*P* < 0.001and ^####^*P* < 0.0001 vs. the PSO control

The biochemical analysis revealed significantly elevated plasma levels of CU in the model group compared to both the PSO and HSO groups. However, PF treatment significantly lowered these factor levels as shown in Fig. 6. In addition, the levels of pyruvic acid in the model group were significantly lower compared to both the PSO and HSO control groups. Treatment with a medium dose of PF resulted in a significant elevation of these factor levels (Fig. 6).

### PF Suppresses NLRP3 inflammasome activation and inflammation in myocardial infarction under high-altitude conditions

**Fig. 7:**
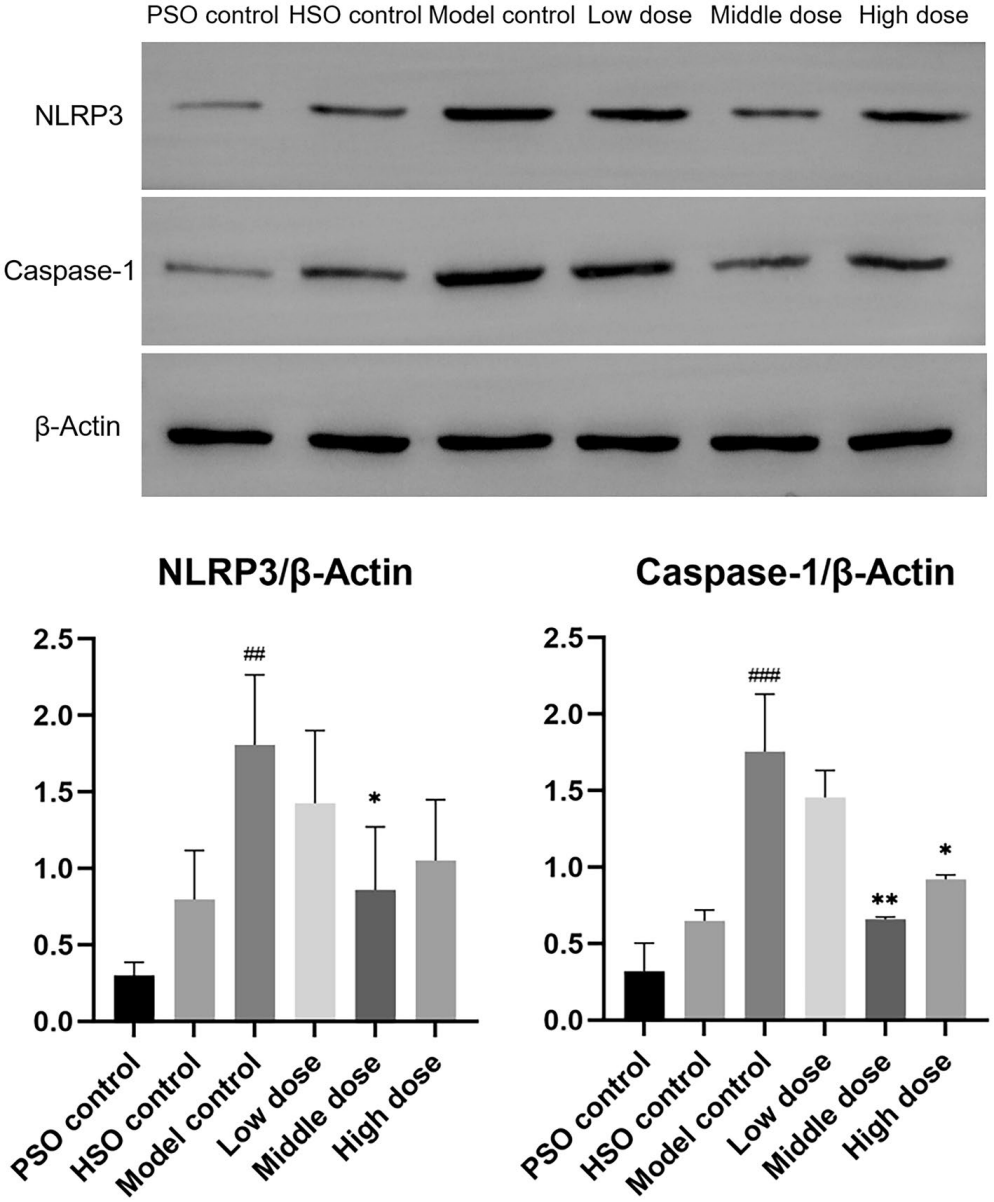
Western blots were performed to assess the protein levels of NLRP3 and Caspase-1, with corresponding statistical analysis presented (*n* = 3). The values represent the means ± SDs. ^*^P < 0.05 and ^**^P < 0.01 vs. the model control; ^##^*P* < 0.01 and ^###^*P* < 0.001 vs. the PSO control

**Fig. 8:**
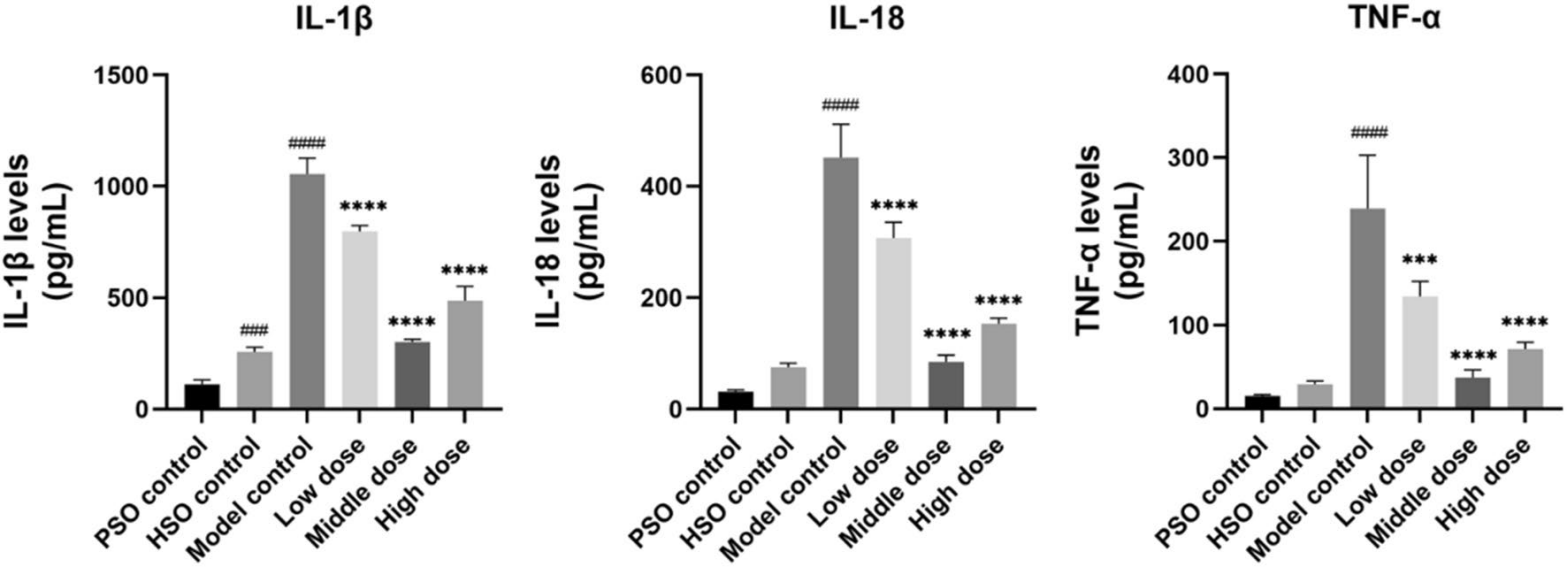
The heart levels of IL-1β, IL-18, and TNF-α, as measured by ELISA (*n* = 6). The values represent the means ± SDs. ^***^P < 0.001 and ^****^P < 0.0001 vs. the model control; ^###^*P* < 0.001and ^####^*P* < 0.0001 vs. the PSO control

To assess the effects of PF on NLRP3 inflammasome-driven inflammation, we measured left ventricular expression of NLRP3 and Caspase-1 by Western blot. PF treatment significantly attenuated myocardial NLRP3 inflammasome activation compared to the model group, as evidenced by reduced NLRP3 and Caspase-1 levels (Fig. 7). The ELISA analysis further revealed that the model group exhibited significantly increased plasma and cardiac levels of the pro-inflammatory cytokines IL-1β, IL-18, and TNF-α in comparison to the control groups. PF treatment markedly decreased circulating and myocardial concentrations of these inflammatory factors (Fig. 8).

### PF attenuates oxidative stress in AMI rats under hypobaric hypoxia

**Fig. 9:**
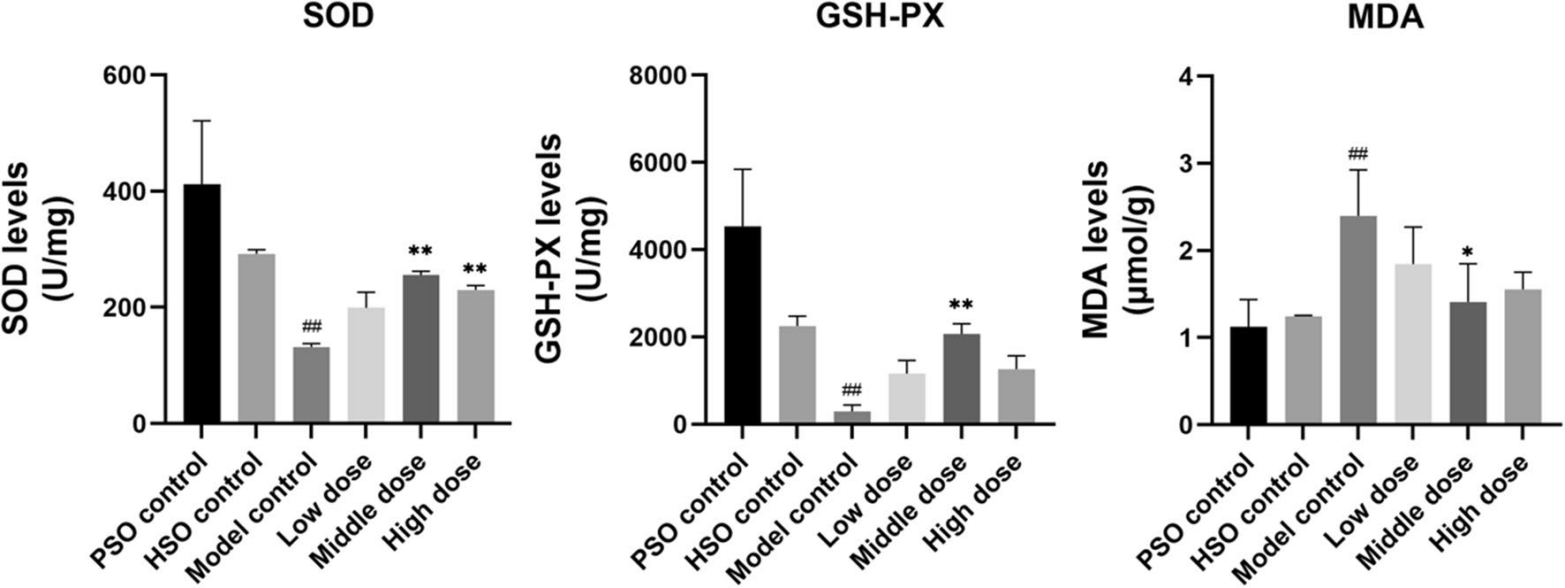
The heart levels of SOD, MDA, and GSH, as measured by ELISA (*n* = 6). The values represent the means ± SDs. ^*^P < 0.05 and ^**^P < 0.01 vs. the model control; ^##^*P* < 0.01 vs. the PSO control

To evaluate the antioxidant effects of PF, we assessed ROS levels in cardiac tissue. PF treatment significantly reduced ROS generation compared to the untreated model group (Fig. 9). It also increased myocardial levels of the endogenous antioxidants SOD and GSH while decreasing malondialdehyde MDA, a marker of oxidative damage.

### PF attenuates cardiac fibrosis and apoptosis in AMI rats under hypobaric hypoxia

**Fig. 10:**
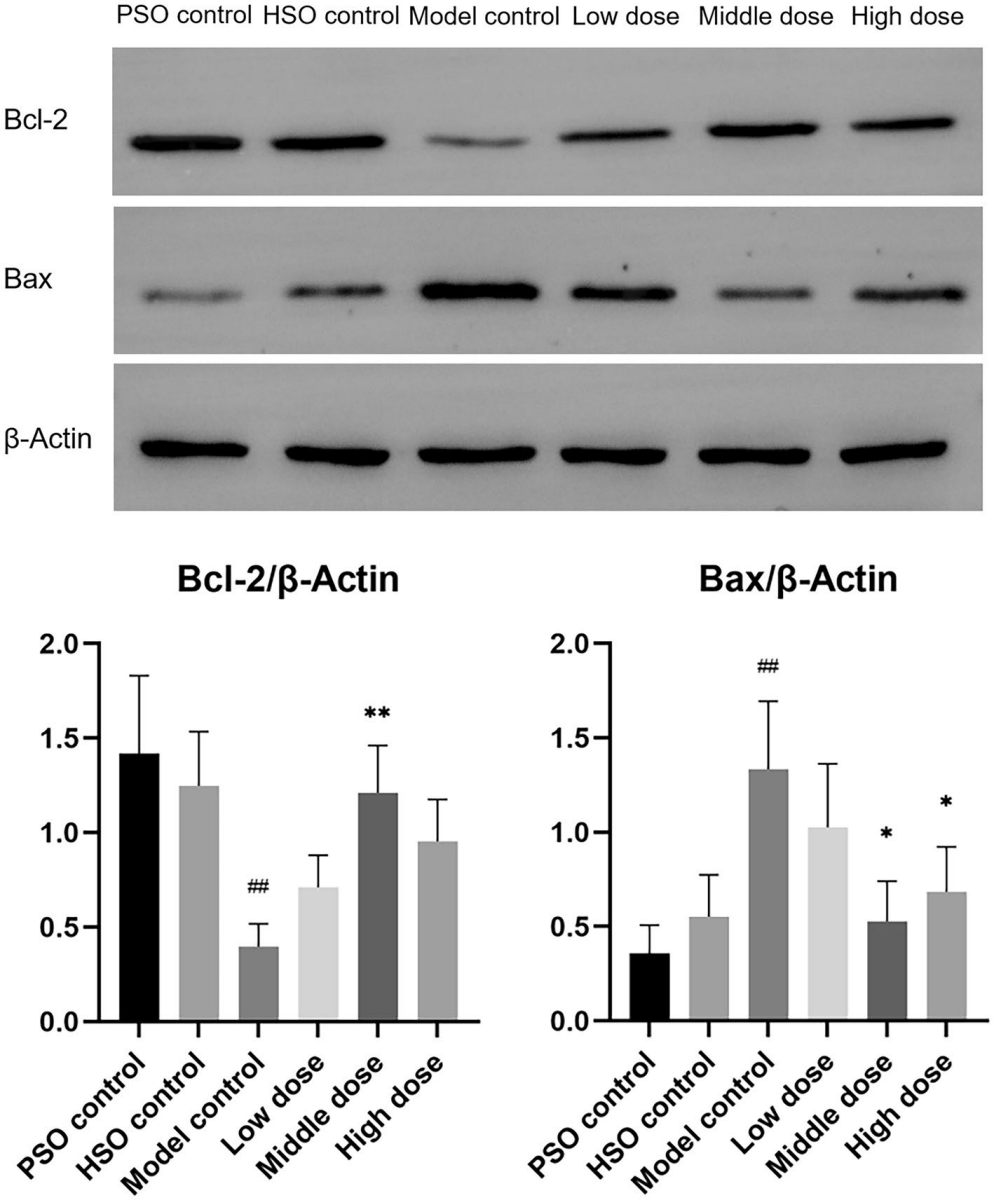
Western blots were performed to assess the protein levels of Bcl-2 and Bax, with corresponding statistical analysis presented (*n* = 3). The values represent the means ± SDs. ^*^P < 0.05 and ^**^P < 0.01 vs. the model control; ^##^*P* < 0.01 vs. the PSO control

**Fig. 11:**
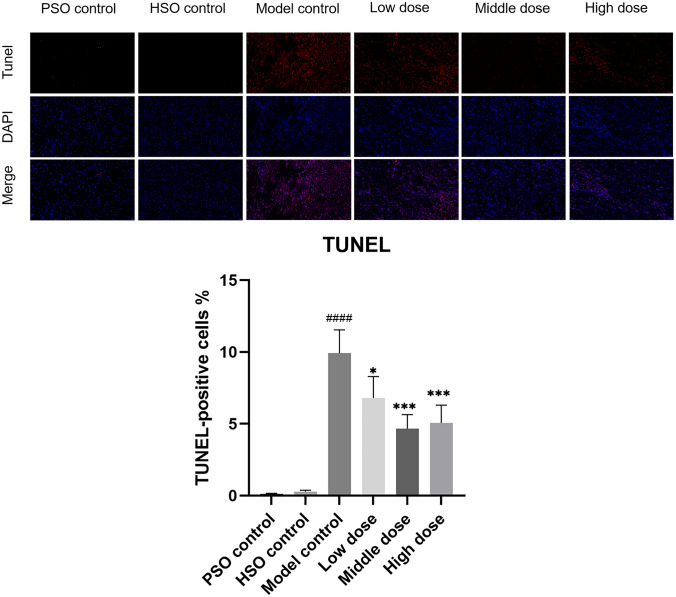
Representative images depicting TUNEL staining patterns in the myocardium were obtained, with corresponding statistical analysis presented (*n* = 6). The values represent the means ± SDs. ^*^P < 0.05 and ^***^P < 0.001 vs. the model control; ^####^*P* < 0.0001 vs. the PSO control

**Fig. 12:**
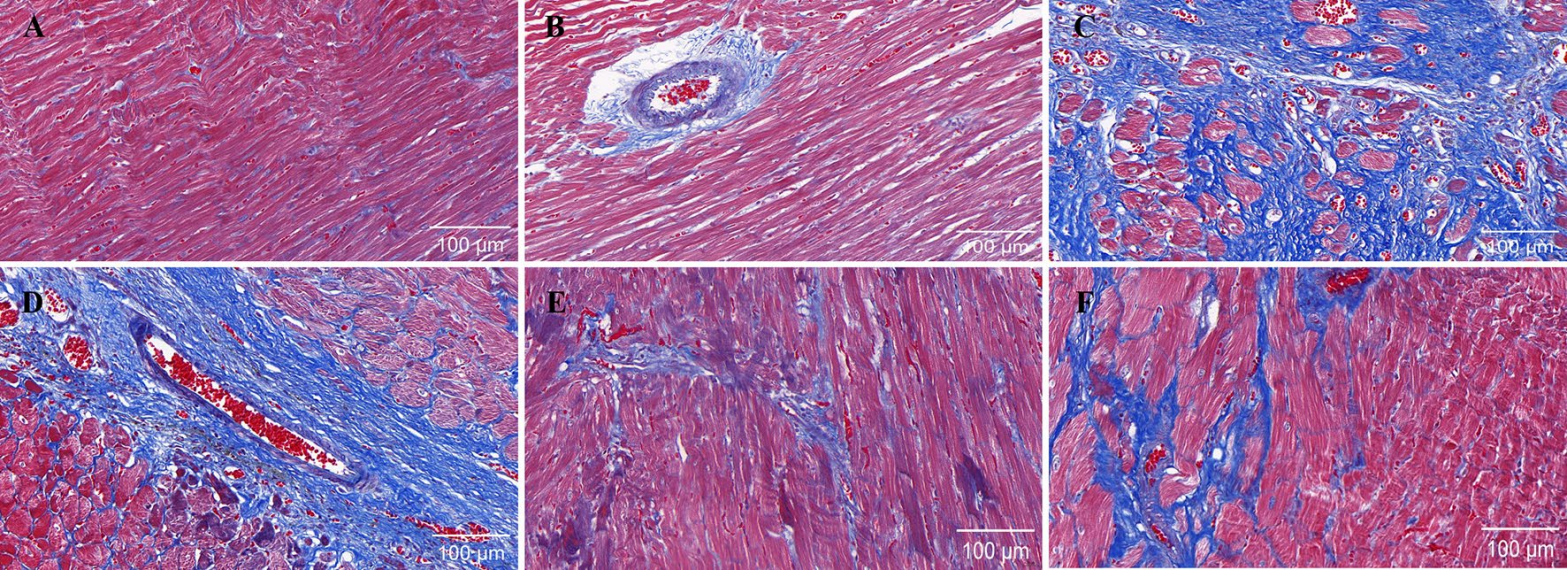
Representative sections of Masson’s trichrome-stained hearts (scale bar: 100 μm): **A** plain sham-operated control; **B** hypobaric hypoxia sham-operated control; **C** model control; **D** low-dose group; **E** middle dose group; **F** high-dose group

We next examined the effects of PF on apoptotic signaling and myocardial fibrosis following AMI under high-altitude conditions. The Western blot analysis revealed a decrease in the expression of the anti-apoptotic protein Bcl-2 and an increase in the expression of the pro-apoptotic protein Bax in the model group compared to rats treated with PF (Fig. 10). TUNEL staining corroborated these results, demonstrating that PF markedly decreased cardiomyocyte apoptosis in the post-myocardial infarction group (Fig. 11). Masson’s staining further revealed severe interstitial and perivascular fibrosis in the untreated infarcted myocardium, which improved with PF treatment (Fig. 12).

## Discussion

In this study, we assessed the cardioprotective effect of PF in a rat model of AMI in a high-altitude environment. CMR evaluation showed that PF treatment significantly attenuated cardiac dysfunction associated with AMI in a high-altitude environment and rats in the PF groups exhibited higher LVEF than the model group. Furthermore, in comparison to the model group, the PF groups exhibited lower LVEDV and LVESV, indicating a mitigation of left ventricular remodeling following acute AMI.

It is worth noting that high-altitude environments can lead to hypoxia, which may exacerbate the impact of copper on the cardiovascular system. Copper is recognized to play a pivotal role in the pathophysiological processes of the cardiovascular system [8]. Importantly, elevated copper concentrations have been associated with heart failure and its prognosis [9, 15]. Recent research demonstrated that chelating excess copper and restoring cardiomyocyte copper transport led to improved mitochondrial and cardiac function in heart failure rats [16].These findings highlight the importance of maintaining proper copper homeostasis to support mitochondrial health in the diseased heart. In our study, serum copper levels were significantly elevated, whereas these changes were mitigated in the PF-treated group, demonstrating enhanced recovery of ventricular function following MI. These findings suggest a potential association between serum copper levels and the progression of adverse left ventricular remodeling following myocardial infarction. Paeoniflorin exhibits the potential to decrease serum copper levels and ameliorate the progression of adverse ventricular remodeling in the infarcted heart.

Dysregulated myocardial energy metabolism significantly contributes to the pathogenesis and progression of heart failure [17]. The study conducted by Tsvetkov et al. revealed that cuproptosis, a recently identified mechanism of cell death, exhibits distinct characteristics compared to apoptosis, pyroptosis, and ferroptosis [18]. The occurrence of cuproptosis is intricately linked to mitochondrial respiration and ATP production, whereas mitochondria are intimately associated with various forms of cell death in heart failure HF, including necrosis, apoptosis, and autophagic cell death [19]. FDX1 serves as an upstream regulator of protein lipoylation and plays a crucial role in mediating copper-induced cell death. Disruption of copper homeostasis can activate FDX1, leading to the detrimental loss of iron-sulfur cluster proteins and subsequent protein stress [18]. In our study, both FDX1 and DLAT expressions increased in the AMI rat model under chronic hypoxia, while their levels significantly lowered in the PF treatment group that showed improved cardiac function, suggesting that FDX1 and DLAT may affect left ventricular remodeling after AMI via copper-mediated cell death, and that PF can reduce the expression of FDX1 and DLAT and thus may be involved in left ventricular remodeling after AMI.

Pyruvate plays a pivotal role in cellular metabolism, particularly in mitochondrial respiration and energy production [20]. In conditions of copper excess, copper can interact with various proteins in mitochondria, affecting their function and leading to disturbances in cellular energy metabolism [21]. Specifically, the activity of FDX1, a critical protein in iron-sulfur cluster formation, is impacted, which in turn affects fatty acid metabolism and the ability of pyruvate to enter mitochondria [22]. Pyruvate, the end product of glycolysis, typically enters the mitochondria where it is converted into acetyl coenzyme A, participating in the citric acid cycle and thus impacting ATP production. In the milieu of cuproptosis, the dysregulated copper may interfere with the normal metabolic pathways of pyruvate, leading to reduced energy production, increased cellular stress, and ultimately promoting cardiomyocyte death [23].In our study, we observed a significant reduction in pyruvate levels in the AMI rat model, which may be attributable to copper-induced mitochondrial dysfunction and disrupted energy metabolism. The recovery of pyruvate levels post-treatment with PF correlated with improved ventricular function, supporting the potential link between cuproptosis, pyruvate levels, and cardiac remodeling.

Apoptosis, in addition to cuproptosis, plays a pivotal role in myocardial remodeling in HF [24]. The balance between the pro-survival protein Bcl-2 and the pro-apoptotic protein Bax plays a pivotal role in governing the intrinsic apoptosis pathway triggered by mitochondrial dysfunction [25, 26]. The preservation of mitochondrial integrity is facilitated by the anti-apoptotic protein Bcl-2, which impedes the release of pro-death factors such as cytochrome c. Conversely, the pro-apoptotic protein Bax promotes permeability in the outer membrane of mitochondria, thereby facilitating the efflux of these apoptogenic factors [27, 28]. Our experimental results showed that PF reduced myocardial apoptosis after AMI by increasing Bcl-2 expression and decreasing Bax expression.

In this study, we have investigated and discussed the cardioprotective properties of Paeoniflorin in a rat model of AMI exposed to hypobaric hypoxia. Understanding the interaction between oxidative stress and hypobaric hypoxia in the context of this research is vital for the development of effective therapeutic approaches for the management of AMI in high-altitude environments. ROS overproduction and inadequate endogenous antioxidant defenses contribute to the development of oxidative stress [29]. Meanwhile, hypobaric hypoxia, the decreased oxygen levels experienced at high altitudes, can exacerbate AMI-caused myocardial injury by inducing oxidative stress. Eduardo et al. discovered that oxidative stress is associated with all high-altitude pathologies, as indicated by increased levels of the biomarker MDA and diminished antioxidant activity of SOD and glutathione peroxidase [30]. In the present study, PF treatment was shown to enhance cardiac function and attenuate ventricular remodeling in rats with AMI exposed to hypobaric hypoxia. Moreover, PF significantly upregulated the expression of key antioxidant enzymes, including SOD and GSH, while concurrently reducing levels of MDA, a reliable biomarker for lipid peroxidation. As a result, PF effectively ameliorated the detrimental impact of oxidative stress on cardiac tissue.

Interaction between cardiac fibroblasts and the extracellular matrix during hypobaric hypoxia is crucial for the onset and development of myocardial fibrosis [31, 32]. Activation of the NLRP3 complex induces caspase-1 activation and subsequent release of the pro-inflammatory cytokines IL-1β and IL-18, thereby eliciting apoptotic signaling cascades that contribute to cardiomyocyte loss and cardiac dysfunction [33]. This can in turn result in ventricular remodeling and eventually heart failure [34–36]. Our study has shown that by reducing the expression of these proteins, treatment with PF can mitigate the left ventricular remodeling induced by AMI under persistent hypobaric hypoxia by counteracting inflammation, myocardial fibrosis, and apoptosis triggered by the NLRP3 inflammasome.

### Limitations

Notwithstanding that this study was conducted using an animal model, whose are satisfactory, further research in the form of clinical trials is warranted to fully validate the cardioprotective effects of PF on humans, particularly under high-altitude conditions. In addition, since this research lasted only 4 weeks, further studies of longer duration spanning from 4 weeks to several months are also warranted for a more comprehensive evaluation of the efficacy and safety of chronic PF treatment. What’s more, the precise mechanisms under the protective effects of PF remain to be fully elucidated. Therefore, additional mechanistic studies related to the underlying mechanism that focus on potential targets, such as oxidative stress pathways, inflammatory cascades, and metabolic alterations are warranted so as to shed light on the underlying processes involved.

## Conclusion

To summarize, this study shows that PF has notable cardioprotective benefits in a rat model of AMI in hypobaric hypoxia conditions, which are often present in high-altitude environments. The beneficial impact of PF on ventricular remodeling is achieved by attenuating inflammation, apoptosis, cardiac fibrosis, and inhibiting cuproptosis and the NLRP3 inflammasome. These findings provide valuable insights into the potential of PF as a promising therapeutic option for treating left ventricular remodeling following AMI, especially in plateau environments. Further investigation is warranted to elucidate the intricate molecular mechanisms underlying the cardioprotective advantages of PF and to evaluate its efficacy and safety in clinical settings.

## Data Availability

All data will be provided upon request to the corresponding author Fabao Gao (gaofabao@wchscu.cn).
